# The effectiveness and health-economic evaluation of “Partner in Balance,” a blended self-management program for early-stage dementia caregivers: study protocol for a cluster-randomized controlled trial

**DOI:** 10.1186/s13063-023-07423-9

**Published:** 2023-06-22

**Authors:** Sander L. Osstyn, Ron Handels, Lizzy M. M. Boots, Sanne C. E. Balvert, Silvia M. A. A. Evers, Marjolein E. de Vugt

**Affiliations:** 1 Department of Psychiatry and Neuropsychology and Alzheimer Center Limburg, School for Mental Health and Neuroscience (MHeNS), Maastricht University, PO Box 6166200 MD, Maastricht, The Netherlands; 2 Department of Clinical Neuropsychology, Vrije Universiteit Amsterdam, Amsterdam, The Netherlands; 3 Department of Health Services Research and Care and Public Health Research Institute (CAPHRI), Maastricht University, PO Box 6166200 MD, Maastricht, The Netherlands; 4 Centre of Economic Evaluations & Machine Learning, Trimbos Institute, Utrecht, The Netherlands Institute of Mental Health and Addiction, Utrecht, The Netherlands

**Keywords:** Dementia, Economic evaluation, Cost-effectiveness, Cost-utility, Online intervention, Cluster RCT, Blended care, Informal care, Self-efficacy

## Abstract

**Background:**

Informal caregivers of people with dementia are crucial in dementia care. However, they are insufficiently supported and report caregiver burdens, which urges the need for cost-effective interventions aimed at supporting caregivers. This paper presents the design of a study evaluating the effectiveness, cost-effectiveness, and cost-utility of a blended self-management program for early-stage dementia caregivers.

**Methods/design:**

A pragmatic, cluster randomized controlled trial with a shared control group will be conducted. Participants will be informal caregivers of people with early-stage dementia and will be recruited by local care professionals. Randomization will be carried out at the level of the care professional level in a ratio of 35% to 65% (control arm vs. intervention arm). Participants in the control arm will receive care as usual and the intervention arm will receive the blended care self-management program “Partner in Balance” within a usual care setting in the Netherlands. Data will be collected at baseline and at 3-, 6-, 12-, and 24-month follow-ups. The primary outcome for effectiveness (part 1) is care management self-efficacy. For the health-economic evaluation (part 2) total care costs and the quality of life for individuals with dementia (cost-effectiveness) and quality-adjusted life years (cost-utility) will be the base case analysis. Secondary outcomes (parts 1 and 2) will include depression, anxiety, perceived informal caregiving stress, service-use self-efficacy, quality of life, caregivers’ gain, and perseverance time. A process evaluation (part 3) will investigate the internal and external validity of the intervention.

**Discussion:**

In this trial, we plan to evaluate the effectiveness, cost-effectiveness, and cost-utility of “Partner in Balance” among informal caregivers of people with dementia. We expect to find a significant increase in care management self-efficacy, and the program to be cost-effective, and provide valuable insights to stakeholders of “Partner in Balance.”

**Trial registration:**

ClinicalTrials.gov, NCT05450146. Registered on 4 November 2022.

**Supplementary Information:**

The online version contains supplementary material available at 10.1186/s13063-023-07423-9.

## Introduction

### Background and rationale

The global economic impact of the total 55 million people living with dementia [1] had an estimated cost of US $1.3 trillion in 2019 [2]. Nearly half of these total dementia care costs were associated with the support and assistance provided by members in the close social environment, known as informal care [2]. In addition to the economic impact, providing informal care to a person with dementia (PwD) negatively influences the caregiver’s general well-being in terms of self-efficacy, caregiver burden, quality of life, and life satisfaction. Caregiving also influences their physical and mental health, more specifically it can lead to stress, anxiety, and depression [3–5]. European healthcare systems insufficiently protect informal caregivers sufficiently against the negative impact of informal care [6, 7]. This is demonstrated by the majority of informal caregivers urging a need for additional support [8]. This unmet need for support for caregivers in combination with the expected increase in dementia’s prevalence and total dementia costs [2] urgently calls for cost-effective interventions aimed at supporting informal caregivers to manage living well at home.

Prior research indicated that non-pharmacological multi-component interventions can reduce caregiver burden [9–11], which is a significant predictor for the institutionalization of the PwD [12, 13]. This was substantiated by reported delayed institutionalization rates in several earlier studies on multi-component interventions [9, 11, 14]. Institutionalization can also be predicted by the health-related quality of life of the caregiver [15]. Prior research demonstrated that similar interventions could effectively target this predictor [9, 16]. Therefore, these interventions could significantly lower total dementia healthcare costs by delaying the institutionalization of the PwD. In addition, informal caregivers’ healthcare usage could also be lowered because it is associated with caregiver depression [17], which is positively influenced by internet-based or technology-based interventions in randomized trials [18, 19]. Therefore, blended e-health interventions consisting of a combination of online therapy and in-person treatment, aimed at informal caregivers, consisting of education, information, and training in self-management skills could potentially be cost-effective due to their efficient use of resources.

The Partner in Balance (PiB) [20] intervention is an online psychoeducation and behavioral modeling intervention for informal caregivers of PwD in the early stage of the disease, coached by a care professional. In earlier research [21], the effectiveness of PiB over an 8-week follow-up period has been demonstrated to significantly improve self-efficacy, mastery, and quality of life compared to usual care in an 8-week randomized controlled trial (RCT) in informal caregivers of persons with mild cognitive impairment (15%), Alzheimer’s dementia (41%) or other dementia types (44%) in the setting of memory clinics and ambulatory mental health clinics [21]. Currently, the effectiveness in the longer term and the cost-effectiveness of the PiB intervention are still unknown. However, this is essential information so that scarce healthcare resources can be utilized as efficiently as possible while maintaining high-quality care that is equal and equitable. This research protocol describes a proposed pragmatic cluster RCT to evaluate the effectiveness (part 1) and to perform a health-economic evaluation (part 2) of the blended care self-management program PiB. In addition, the method for a process evaluation (part 3) will be described.

### Objectives

This proposed research consists of three parts: (1) an effectiveness analysis, (2) a health-economic evaluation, and (3) a process evaluation.

#### Part 1: effectiveness

The primary objective is to estimate the effectiveness of the blended care program PiB compared to usual care over 12 months. We hypothesize that the care management self-efficacy in terms of CSES of caregivers who receive PiB will have a significantly better self-efficacy than that of caregivers who only receive usual care. The secondary objective is to explore the effect of PiB on the primary outcome care management self-efficacy at 24-month follow-up and on the secondary outcomes: perseverance time, quality of life, service-use self-efficacy, positive experiences related to informed care, experienced care burden, anxiety, caregiver gains, and depression up to 24-month follow-up.

#### Part 2: health-economic evaluation

The health-economic evaluation consists of two primary objectives: (1) to estimate the within-trial cost-utility and uncertainty of PiB compared to usual care from a societal perspective over a time horizon of 12 months using EQ5D5L-based quality-adjusted life years and care use from both caregiver and PwD summed, and (2) to conduct a budget impact analysis. We expect PiB to be cost-effective. In addition, the health-economic evaluation includes the three following secondary objectives: (1) to estimate the within-trial cost-effectiveness and uncertainty of PiB compared to usual care from a societal perspective over a time horizon of 12 months, with effectiveness measured in terms of QOL-AD; (2) to perform a cost-consequence analysis to describe all relevant health outcomes, quality of life, and disaggregated costs categories; (3) to explore the health-economic outcomes of PiB over 24 months; and (4) to estimate the lifetime cost-utility and uncertainty of PiB compared to usual care from a societal perspective using a decision-analytic model with extrapolated trial effects.

#### Part 3: process evaluation

A process evaluation aims to evaluate the internal and external validity of this research. This will be attempted by monitoring the research’s sampling process, and the intervention quality (intervention adherence and the experiences of persons with dementia, caregivers, and care professionals with PiB).

## Methods/design

**Fig. 1:**
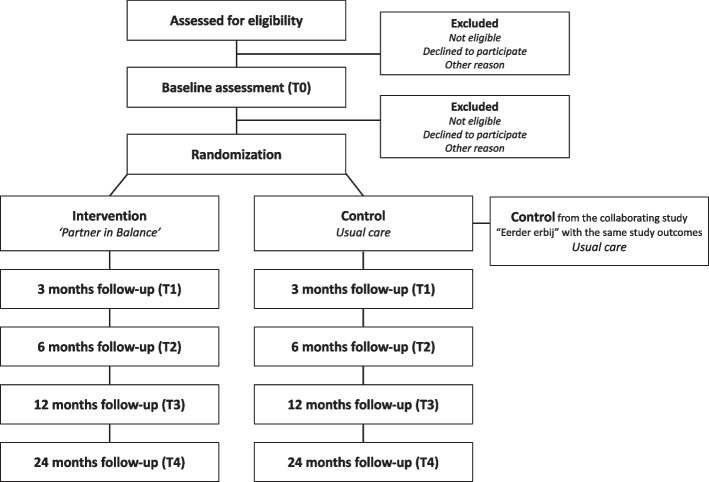
Participant flow diagram according to Consolidated Standards of Reporting Trials (CONSORT)

A pragmatic, cluster RCT design will be used to study participants’ longitudinal outcomes at baseline (T_0_), at 3 months (T_1_), 6 months (T_2_), and 12 months (T_3_) follow-up, and partly at the 24-month follow-up (T_4_), in the control and intervention arm. The control arm will be shared with another collaborating study (called “Eerder-Erbij”) at the Vrije Universiteit of Amsterdam (the Netherlands). Concretely, this entails that the two studies share their recruitment efforts by each supplying part of the targeted control arm participants but recruit their own intervention arm participants to compare their respective interventions to the shared control arm. The collaborating study will apply the same inclusion criteria, same study procedures, and evaluate the same outcomes over time. See Fig. 1 for the participant's flow diagram of the research. This study protocol follows the Standard Protocol Items: Recommendations for Interventional Trials (SPIRIT) guidelines [22] (see Additional file 1).

### Study setting

Study participants will be recruited in primary care by their local care professionals (e.g., case managers dementia, and home nurses) in the Netherlands.

### Eligibility criteria

In order to be eligible to participate in this study, a participant must meet all of the following inclusion criteria: (1) the subject is an informal caregiver of a person with early-stage dementia; (2) the PwD, for whom they care, has a diagnosis of dementia (self-reported or known by the recruiting organization) or underdiagnosed dementia (no formal diagnosis but symptoms of dementia, as judged by the recruiting care professional, based on their professional opinion and experience); and (3) the PwD is not yet receiving formal care related to personal activities of daily living (ADL) on account of his/her dementia more than once a week (defined by receiving assistance from a paid worker (e.g., health or social care worker) such as help with dressing/undressing, washing/bathing/showering, toileting, feeding/drinking, taking medication or attending day activity or daycare center).

An informal caregiver who meets any of the following criteria will be excluded: (1) participating in another trial with similar objectives as this research; (2) younger than 18 years; (3) no basic internet skills; (4) no access to the internet at home; (5) already received the PiB program; (6) receiving a similar support program; and (7) not able to follow COVID19 instructions. The subject will also be excluded from participation if the informal caregiver or the PwD: (1) has a major mental or physical illness; (2) does not have a minimum understanding of the Dutch language; or (3) the dementia of the PwD is caused by human immunodeficiency virus (HIV), acquired brain impairment, down syndrome, chorea associated with Huntington’s disease, or alcohol abuse.

### Recruitment

Study participants will be recruited through care professionals with ≥ 1 year of experience with the target population and no prior experience with PiB who are already in contact with the targeted study population (i.e., informal caregivers of a person with early-stage dementia). These care professionals will be recruited from several home care organizations within the Netherlands via the Dutch Dementia network (https://www.dementienetwerknederland.nl/) and via contacts of the Alzheimer Center Limburg. Participating professionals will be asked to screen their caseload for about 1–5 eligible informal caregivers, provide them with introductory information on PiB and, if are interested, send their contact details to the research team. The potential study participants will then be called by the research team and, if they are interested, receive a study information letter and study consent form. Potential participants will be contacted again within 2 weeks. When the caregivers are willing to participate, they will be screened during a short telephone interview to check the inclusion and exclusion criteria. Participation starts after the research team has received written informed consent from the informal caregiver. Participants will be asked separately for consent to reuse their data for future research (participation is thus possible without consent for data reuse).

### Randomization

Before randomization, the participant will complete the baseline assessment. Then, cluster randomization on the level of the care professionals will be applied. Therefore, professionals and their corresponding set of 1–5 recruited participants will be randomly allocated either to the intervention arm (receives the PiB program in addition to usual care) or to the control arm (only receives usual care) by a spreadsheet random number generator. Because of the shared control arm design, fewer control arm participants need to be recruited than intervention participants. Therefore, in this study, 65% of participants will be allocated to the intervention arm and 35% to the control arm. Stratified randomization [23] will be utilized to ensure that distribution among care professionals within each organization will be as even as possible. Finally, due to variations in the cluster sizes, deviations could occur for the target sample size. In case the allocation diversion is larger than 10% than the desired allocation of 65/35 (e.g., 77% was allocated to the intervention arm at some point during recruitment), the probability to be allocated to the under-allocated arm (e.g., the control arm) will be set to 0.80. The randomization process, enrollment of participants, and assignment of participants to their arms will be executed by the main researcher (SLO). Due to the nature of the intervention in this research, blinding of the participant, care professional, outcome assessor, and data analyst will not be attempted because of pragmatic limitations.

#### Matching

To minimize any selection bias because of the shared control group with the collaborating study, control participants will be matched. For the matching, an additional 15 control participants will be recruited to allow for a larger pool of control participants to match from. The best-fitted controls from the collaborating study will be included in our control arm to reach the targeted sample size of the control arm. Matching will be done on the following characteristics: care organization (size and type); care professional (type and educational level); participant informal caregiver (age, sex, educational level, and informal care hours); PwD (age, sex, educational level, and dementia severity); and the preferences, beliefs, and experiences of e-health or face-to-face contact of the informal caregivers and professionals. Additionally, propensity scores will be calculated based on the questions handling restraint of the informal caregivers due to COVID-19.

### Intervention

#### Intervention condition

Participants in the intervention arm will receive care as usual as well as the 8-to-12-week PiB intervention [20]. The program was developed with informal caregivers and care professionals at the Alzheimer Centre Limburg (ACL). The self-management program consists of a face-to-face intake session, an online period, and a face-to-face evaluation session with a personal coach. Coaches are care professionals who priorly received a 3-h training in self-management techniques, goal setting, and online help. The development of the intervention is described in detail elsewhere [20, 21]. In summary, the aim of the intervention is for informal caregivers to increase caregiver resilience to avoid future caregiver burdens. In the intake session, the participants will be familiarized with the program, set goals that they wish to accomplish through their participation, and select four out of the twelve available modules (based on their personal needs and areas of interest). The module themes are provided in Table 1. Each module consists of (1) a short video with experiences/tips from peers, (2) psychoeducation (information) combined with written examples and tips from peers, (3) a reflective assignment, and (4) a 5-step self-management plan. Following the intake, the informal caregivers will complete their selected modules online over 8 to 12 weeks. For each module of the four modules, 2 to 3 weeks are allocated, but participants will be allowed to complete the modules at their own pace because of the self-management principle. After the online period, participants will meet face-to-face with their personal coach to discuss whether their ability to cope with future difficulties and accomplish goals has been improved in an evaluation session. Throughout the intervention, participants are guided by a personal coach, whose main tasks are supporting participants in choosing modules that fit their situation, helping participants identify feasible goals, offering techniques to achieve goals, and providing participants with general constructive feedback on their assignments within the modules. Additionally, they also monitor the completion or incompletion of the assignments within the modules frequently, thus improving intervention adherence.

**Table 1:** Themes of the available “Partner in Balance” modules

Module theme	
1	Acceptance
2	Balance in activities
3	Communication with family member and environment
4	Coping with stress
5	Focusing on the positive
6	Insecurities and rumination
7	Self-understanding
8	The changing family member
9	Social relations and support
10	Impact on family life
11	Sexuality and intimacy
12	Combining care with work

This study is a pragmatic trial. Therefore, during the follow-up period other interventions and concomitant care is allowed for participants and will be monitored. The intervention will be provided individually and discontinued or modified if considered necessary by the care professional or participant.

#### Control condition

Participants in the control arm will not receive the PiB intervention but will continue to receive care as usual. More information on usual care in the Netherlands can be consulted in the Dutch guidelines for dementia care [24]. However, in the Netherlands, common dementia care practice varies between regions, but most community-dwelling PwD are cared for by informal caregivers in their own homes. The control arm participants are guided by a dementia case manager. These are trained professionals who visit on fixed moments and offer emotional guidance, help arrange care, and help with navigating the Dutch healthcare environment [25].

### Procedure

The informal caregivers in the intervention and control arm will be assessed at five-time points: baseline assessment (T_0_), after 3 months (T_1_), 6 months (T_2_), 12 months (T_3_), and 24 months (T_4_). The informal caregivers will complete all measurements either via an online survey, by paper post, or via a telephone interview with the researcher depending on their preference. An overview of the enrollment and assessment procedure is shown in Table 2. To ensure the quality of the research, several measures are taken to limit missing data during the conduct of the trial based on recommendations by Little and colleagues [26]. An example was limiting the data collection burden and inconvenience for participants in the development and selection of the questionnaires.

**Table 2:**
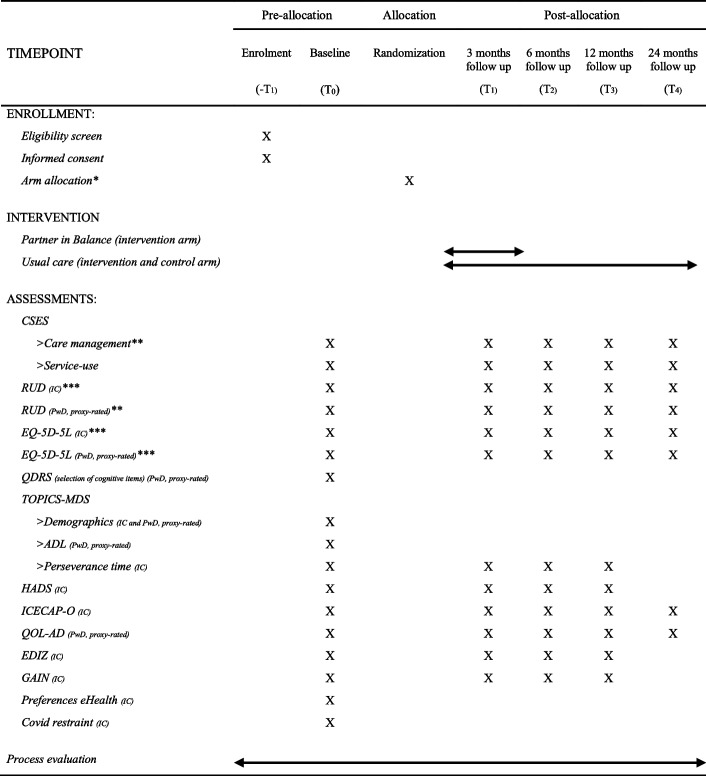
Schedule of enrolment and assessments according to Standard Protocol Items: Recommendations for Interventional Trials (SPIRIT)

*Abbreviations*: *CSES* Caregiver Self-Efficacy Scale, *RUD* Resource Utilization in Dementia, *EQ-5D-5L* EuroQol-5 Dimension Five Levels, *QDRS* Quick Dementia Rating System, *TOPICS-MDS* The Older Persons and Informal Caregivers Survey Minimum DataSet, *HADS* Hospital and Anxiety Depression Scale, *ICECAP-O* Investigating Choice Experiments for the Preferences of Older People, *QOL-AD* Quality of Life in Alzheimer’s Disease, *EDIZ* Ervaren Druk door Informele Zorg, *GAIN* Gain in Alzheimer care Instrument, *IC* Informal caregiver, *PwD* Person with dementia

*Random cluster allocation on the level of the care professional takes place after the completion of the baseline assessment

**Primary outcomes for part 1 (effectiveness)

***Primary outcomes for part 2 (health-economic evaluation)

### Outcomes/measurements

#### Demographics

Demographic characteristics, including age, sex, and educational level of both the caregiver and PwD, will be obtained using a selection of The Older Persons and Informal Caregivers Survey Minimum DataSet (TOPICS-MDS) [27]. This standardized questionnaire measures the general health condition of people with dementia and their informal caregiver. There is a version for the caregiver and for the care receiver.

Demographics of the care professional, such as age, sex, educational level, function, experience with the target group, and care model/style-related characteristics, will be collected using a self-developed questionnaire. In addition, various characteristics of the care organization will also be requested.

#### Primary outcome measures

The primary effectiveness (part 1) outcome is care management self-efficacy which will be measured via the care management self-efficacy domain of the Caregiver Self-Efficacy Scale (CSES) [28]. The domain contains five items that are scored self-rated from 1 (not at all certain) to 10 (very certain). In earlier research, good reliability was demonstrated for the Dutch version of the CSES [21].

For the health-economic evaluation (part 2), the primary outcomes are total care costs and health-related quality-of-life of the informal caregivers and the PwD taken together. The total care use will be obtained using the Resource Utilization in Dementia (RUD) [29]. This assessment measures the care resource use by the informal caregiver (self-rated) and the PwD (proxy-rated). For the caregiver, these resources include informal caregiver time (personal and instrumental activities of daily living and supervision), visits to care professionals, hospitalization, and productivity loss for those with a paid job. The questions on medication usage were not included in this study. For the PwD, it covers accommodation (intermediate form, dementia-specific residential or long-term institutional care), visits to care professionals, hospitalizations, and social care services.

Health-related quality of life of the informal caregiver (self-rated) and the PwD (proxy-rated) will be determined via the EQ-5D-5L assessment developed by EuroQol [30], which measures health-related quality of life on five dimensions: mobility, self-care, usual activities, pain/discomfort, and anxiety/depression on a five-point Likert scale. This scale is recommended as a generic preference-based scale for health-economic analysis in the Dutch guideline for health-economic evaluation [31].

#### Secondary outcome measures

The Hospital and Anxiety Depression Scale (HADS) [32] provides separate scores for depression and anxiety, each with 7 items self-rated on a four-point Likert scale ranging from 0 (not at all) to 3 (a great deal of the time). Total scores range from 0 to 21 per topic, with higher scores indicating more anxiety or depression levels for the informal caregiver. Good reliability was detected in previous research [33].

The Perceived Stress by informal caregiving (EDIZ) (“Ervaren Druk door Informele Zorg”) [34] is a 9-item measurement for informal caregivers to assess self-perceived pressure from informal care. The items are scored on a five-point Likert scale ranging from “no!” to “yes!.” Although the assessment is not as widely applied as the Zarit burden interview (ZBI), the EDIZ was chosen because it contains fewer emotionally sensitive items that might lead to drop-out.

Service-use self-efficacy will be measured via the service-use self-efficacy domain of the Caregiver Self-Efficacy Scale (CSES) [28]. Just as the care management domain, which is used as a primary outcome, this domain contains five items that are scored from 1 (not at all certain) to 10 (very certain).

The Investigating Choice Experiments for the Preferences of Older People (ICECAP-O) [35] measures five important capability attributes of quality of life: attachment, security, role, enjoyment, and control. All items are scored from 1 (no capability) to 4 (full capability), with higher scores indicating a higher overall quality of life for the informal caregiver. The scale has been translated into Dutch [36]. Previous research has indicated that this instrument may be more sensitive to differences between interventions and comparators and that it measures broader outcomes than the EuroQol five dimensions questionnaire [37].

Caregivers’ gain will be measured using the Gain in Alzheimer care Instrument (GAIN) [38]. A total of 10 items are scored on a five-point Likert scale ranging from “disagree a lot” to “agree a lot.” The total summed score ranges from 0 to 40, with higher scores indicating that the caregiver has gained more in dementia caregiving. Previous research has indicated that the GAIN is both reliable and valid among caregivers of community-dwelling PwD [38].

Part of the TOPICS-MDS is perseverance time, which measures how long an informal caregiver is indicated to maintain current care if the caregiving situation remains unchanged. It is rated on a six-point Likert scale ranging from “less than a week” to “more than two years” [27].

The Quality of Life in Alzheimer’s Disease scale (QOL-AD) [39] measures the quality of life for individuals with dementia on 13 separate items on a four-point Likert scale ranging from “poor” to “excellent” and will be proxy-rated. This questionnaire showed adequate reliability in previous research [40].

The execution and independence of the instrumental ADL of the PwD will be proxy-rated through the usage of a selection of the TOPICS-MDS [27].

The Quick Dementia Rating System (QDRS) [41] measures cognitive impairment for people with dementia and consists of six behavioral and four cognitive questions though, in this research, only the latter will be included proxy rated. These four items are scored on a scale from 0 to 3, with higher scores indicating more cognitive impairment. A previous study showed good reliability [41]. In combination with the instrumental ADL measures from the TOPICS-MDS, this will enable a relatively detailed judgment of the severity of dementia.

A self-developed questionnaire will obtain the informal caregivers’ and professionals’ preferences, beliefs, and experiences of e-health or face-to-face contact.

COVID-19 restraint of the informal caregiver will be measured using a self-developed questionnaire. Informal caregivers will answer two statements on a five-point Likert scale ranging from “totally agree” to “completely disagree.”

Care professionals will be asked to keep a logbook on how much time they spent on coaching-related activities (administration, intake session, providing online feedback, and evaluation session) to calculate the costs of the PiB intervention.

### Data management

Data from the assessments will be collected in password-protected electronic case report forms (eCRFs). To promote data quality, all data will be manually checked by a researcher (SLO) for completeness and plausibility shortly after it is received. Missing data or data that does not fall within a plausible range will be verified with the participant via telephone. After the research, the data will be archived in the DISQOVER data repository at Maastricht University.

### Confidentiality

Personal data and measurement data will be stored separately and according to current standards for data security and data privacy. All personal data of potential and enrolled participants are password protected and can only be accessed by authorized persons. Paper documents (e.g., informed consents or paper questionnaires) will be stored in a secure way.

### Sample size

The sample size calculation was based on an earlier short-term effectiveness study of PiB [21] that targeted a similar population and used the CSES as a primary effectiveness outcome measure. With an assumed cluster size of 10 informal caregivers, alpha 0.05, power 0.80, and 25% drop-out at 6 months, the total sample size is 126 participants (with 63 in the control arm and 63 in the intervention arm). However, the sample size will be recalculated based on the CSES after 52 participants have completed their baseline assessment so that the targeted sample size can be adjusted with updated information on the standard deviation.

### Statistical analyses

All analyses will be carried out using STATA® version 17.0 for Mac according to the intention-to-treat (ITT) principle unless specified otherwise. In this study, we defined ITT as the subsample of a person with a baseline assessment and at least one observation on the primary effectiveness outcome measure or the primary health-economic outcome measure.

#### Part 1: effectiveness

Prior to the analysis, the data will be examined for missing data and described. To verify the randomization procedure, potential differences in the baseline characteristics of the intervention and control arm will be examined using t-tests for continuous variables and the chi-squared (*X*^2^) test for categorical variables. A mixed generalized linear model (GLM) will be fitted to the care management CSES score (dependent variable) based on the recommended model of Bell and colleagues [42]. The model will include the following independent variables: trial arm, time (categorically), and the interaction between the trial arm and time. Time will be treated as a categorical variable because Donohue and Aisen [43] discuss that data from early in the trial has less influence in a categorical time model framework. As PIB is aimed at sustainable effects over a longer period, we are interested in the outcome at the end of the trial. Therefore, we believe this approach fits better than treating time as continuous. A link function and distribution family will be chosen that best fits the data (in terms of normality of residuals and linearity). A random intercept will be employed to acknowledge the hierarchical structure of the data (observations clustered within participants, and participants clustered within professionals). An unstructured covariance matrix for residual errors will be applied. If the p-value related to the intervention coefficient is less than 0.05 (2-sided) the intervention will be considered to have a statistically significant effect on the primary outcome, which would confirm our hypothesis. For this analysis, any missing data will be assumed missing at random (MAR) and handled by the mixed model.

#### Part 2: health-economic evaluation

##### Cost-utility and cost-effectiveness analysis

The health-economic evaluation will be conducted from a societal perspective with a time horizon of 12 months. In the base case analysis, both an incremental cost-utility ratio (ICUR) and an incremental cost-effectiveness ratio (ICER) will be calculated for the cost-utility and cost-effectiveness analysis respectively. The ICUR will be estimated as the total societal costs difference (both informal caregiver and PwD) divided by the sum of the difference in informal caregiver EQ-5D-5L-based quality-adjusted life-years (QALYs) and PwD proxy-rated EQ-5D-5L-based QALYs between the intervention and control arm. The ICER will be estimated as the difference in total societal costs between the intervention and the control arm and will be divided by the difference in effect in terms of QOL-AD change from the baseline score between the intervention and the control arm.

Total costs will be calculated in Euros (€) from a societal perspective and will include intervention and healthcare costs. The costs will be calculated by multiplying each care use outcome from the RUD instrument by its unit price, over the 12-month period (baseline to the last follow-up measure). Unit prices will be based on the Dutch guidelines for cost calculations in healthcare [31] or other sources if not included in the guidelines. The friction cost approach will be used to estimate the lost productivity costs of the informal caregiver, using the measurements of productivity loss as obtained by the RUD. During the analysis, discounting will be applied for the 12-month follow-up. The discount rates will be based on the Dutch cost guidelines with 4% for costs and 1.5% for QALY’s [31]. Finally, as the recall period of most items of the RUD instrument is shorter than the time between measurements, linear interpolation will be used between cost estimates at the follow-up moments. The intervention costs will be calculated based on the estimated costs for (1) the PiB coach training for professionals, (2) the coaching activities of the professional, and (3) the PiB license costs of €200 for each participant (which covers all other intervention costs, e.g., PiB IT system). To calculate the costs for the training and coaching activities, the standard unit prices for the professional will be multiplied by the time required for the training and the mean time spent on all coaching and training activities during the trial.

To calculate the QALYs, the health conditions derived from the EQ-5D-5L will be expressed in utility scores. The Dutch value set for utility scores [44] will be adopted. Hereafter, the QALYs will be calculated by weighing the length of the time spent in a particular health state by the utility at each measurement point, using linear interpolation between measurements.

To handle the uncertainty surrounding the ICUR and the ICER, bootstrapping with 5000 replications will be used to estimate 95% bootstrap intervals around cost and QALY differences. The incremental (difference between control and intervention) outcome (12-month QOL-AD effect, total QALYs, and total costs) will be estimated using a mixed GLM using the outcome as the dependent variable, and trial arm and baseline value (baseline QOL-AD, baseline utility, and baseline 3-month costs, respectively) as independent variables. A random intercept will be employed to acknowledge the hierarchical structure of the data (observations clustered within professionals). Uncertainty surrounding the ICUR and ICER will be graphically presented on a cost-effectiveness plane. A cost-effectiveness acceptability curve will show the probability that the intervention is cost-effective in comparison with usual care for a range of willingness-to-pay values.

Missing data will be handled using the guidance from Faria and colleagues [45]. Any missing follow-up data will be tested for conditionality of observed factors at baseline and missingness status at each observation will be tested for conditionality on observed factors at the previous observation. If missingness is only conditional on baseline factors, baseline missing is limited and missingness is not disaggregated (e.g., mostly all or none of the scales are missing at an observation), it will be handled using inverse probability of censoring weights. If missingness is conditional on previous observation it will be handled using multiple imputations.

##### Decision-analytic model

An illness-death Markov model will be used to simulate the lifetime cost-effectiveness related to the institutionalization of the person with dementia over a lifetime period. The model will be developed by combining the results from the trial with published estimates on quality of life, care cost, and mortality in dementia. If institutionalization occurs frequently during the trial follow-up period, a parametric survival model will be fitted to the institutionalization data in the control and intervention arm, and the survival model will be used to extrapolate institutionalization over a lifetime. If insufficient observations, a published Dutch institutionalization rate will be multiplied by published relative risks of caregiver outcomes on institutionalization (such as caregiver burden or perseverance time) to the power of observed trial effect on those caregiver outcomes. This will provide a plausible translation of the caregiver outcomes to prevent the institutionalization of the person with dementia.

##### Budget impact analysis

A budget impact analysis will be performed based on the recommendations from Sullivan et al. [46] In the budget impact analysis, the effectiveness of the intervention will be extrapolated over 3 years. Perspectives that will be considered are the societal and the government (in the Netherlands: Budget Kader Zorg) perspectives. Different implementation scenarios (ranging from 0 to 100% implementation) will be evaluated. The total number of participants eligible for the intervention will be estimated based on Dutch epidemiological data. Resource utilization will be calculated by multiplying the number of eligible participants with the resource utilization rates obtained from the health-economic evaluation. Different prices will be used to value resource use depending on the perspective of the analysis: Dutch standard costs from the societal perspective and actual Nationaal Zorg Autoriteit (NZA) tariffs from the government perspective. Both resource use and annual costs will be presented over a 3-year period for all perspectives. Aggregated and disaggregated (e.g., medical care, secondary care, and productivity losses) total costs per year will be presented for the different perspectives and scenarios.

#### Process evaluation (part 3)

In addition to the cluster RCT, a process evaluation will be performed to evaluate the internal and external validity. In a previous study on PiB, an extensive process evaluation has been conducted [47]. Therefore, to avoid overburdening participants, a shortened version of that process evaluation will be used to evaluate the intervention and sampling quality.

The sampling quality will be evaluated via the descriptions of (1) the recruitment procedure, (2) the informed consent procedure, (3) the allocation and randomization procedures, and (4) the methodology of the shared control arm. This data will be obtained from the research database. Additionally, the experienced facilitators and barriers to the recruitment of informal caregivers will be collected via a self-developed online survey completed by the care professionals involved in participant recruitment. Finally, the reach will be calculated by comparing the number of participating informal caregivers to the total number of approached individuals.

For the intervention quality, (1) general satisfaction and (2) treatment fidelity will be evaluated. The general satisfaction will be collected via a self-developed online questionnaire, which caregivers and care professionals will be asked to complete after the eight-week intervention. Treatment fidelity will be collected via a self-developed questionnaire for the informal caregivers and care professionals which will ascertain the extent to which they were able to follow or provide the intervention as planned. This will be combined with website monitoring in the form of clickstreams to determine the complete treatment fidelity of the coach and user. Finally, based on a recommendation to enhance treatment fidelity [48], all informal caregivers will be asked at all measuring moments whether they had received a similar caregiver support program in the past year to ensure that no participants received a similar intervention during the trial duration that might have influenced their outcomes.

### Data monitoring and adverse events

The Medical Ethics Review Committee of Maastricht University stated that the study does not need a full review according to the Dutch Medical Research with Human Subjects Law (WMO) which means that the study brings a very low health risk for participants. For this reason, a data monitoring committee will not be needed for this trial, and adverse events will not be formally monitored. However, information on death, hospitalization, and institutionalization is structurally collected and will provide insight into these serious adverse events.

### Protocol modifications

Protocol amendments and relevant changes will be communicated to the local ethical committee of the University of Maastricht and the sponsor. Additionally, these changes will also be updated in the trial registration (ClinicalTrials.gov, NCT05450146).

### Dissemination plans

The results from the trial will be shared with the scientific community by publishing the study results in international peer-reviewed open-access scientific journals and updating the trial registration (ClinicalTrials.gov, NCT05450146). In addition, the results will be communicated with the trial sponsor, study participants, care professionals, health service providers, and the dementia networks in the Netherlands. For future articles, the International Committee of Medical Journal Editors (ICMJE) guidelines [49] will be used for authorship eligibility and there is no intended use of professional writers. After the research, the research syntaxes and an anonymized version of the data will be made available for reuse upon reasonable request.

## Discussion

In this paper, the design of a cluster RCT was described aimed to evaluate the effectiveness and cost-utility/cost-effectiveness of the PIB blended care program for informal caregivers of PwD.

During the preparation of the trial, several alternative study designs have been considered by the research team, mainly in terms of their validity and practicality. A stepped-wedge design would probably have increased willingness to participate because all participants would have received the PiB intervention. However, it was not preferred due to the long follow-up period leading to participants of the control arm to no longer being in the targeted early dementia stage when starting the PiB program. In addition, a long delay in the treatment time after inclusion could have significantly reduced power [50], and a stepped-wedge design was considered impractical considering the shared control arm. A three-arm randomization design (control arm, PiB intervention arm, and intervention arm from the collaborating study) was considered but not adopted because we expected professionals to prefer either the PIB intervention or the collaborating study intervention. If a professional is allocated to a (strongly) unpreferred intervention, this could negatively impact recruitment and the intervention’s performance. Finally, an RCT with randomization at the individual level was considered for its gold standard in research. However, it was disregarded because of its risk of contamination [51, 52], which would negatively affect the reliability and validity of the study [53]. This contamination could occur when a professional trained for the PiB intervention also provides care for a participant in the control arm. After considering these designs, the research team concluded that a cluster-randomized controlled trial design was the optimal balance between validity and practicality.

For trials with cluster randomization, the potential for selection bias is high because the allocation to treatment is usually predetermined for each member of the cluster, which is a common but undesired practice [53, 54]. This could be demonstrated when a care professional who has foreknowledge of their allocation to the PiB intervention arm likely approaches potential participants in a different way than a care professional who known has been allocated to the control arm. Therefore, in this study, clusters will be randomized after inclusion and baseline measurement to eliminate foreknowledge that can lead to selection bias. During the randomization process, stratified randomization will be used to limit potential selection bias due to a possible association between the organization and the impact of the care received by the informal caregiver [23].

The shared control group will increase the feasibility of the recruitment of participants, which is often a serious challenge in research. However, a shared control group also brings a risk of selection bias. We expect that the participants in the collaborating study are a slightly different selection of the same target population for the following reasons: (1) they will be recruited through a different type of collaborating organizations and (2) they might have different participation considerations for the collaborating study, considering it will be a peer-based group intervention. To prevent this selection bias, matching will be executed to control for these anticipated differences.

The study may yield some limitations. A limitation is that the PiB intervention is only aimed at informal caregivers with internet skills. Therefore, the results of this study will not be generalizable to all informal caregivers. Another limitation is that, due to the nature of the intervention, participant and care professional blinding was considered not feasible by the research team. The absence of blinding could lead to a placebo effect.

In conclusion, the results of this research will add to the scientific knowledge of the effectiveness of the blended care intervention “Partner in Balance.” The program is hypothesized to improve informal caregivers’ self-efficacy and is expected to be cost-effective. This study will provide valuable insights to informal caregivers, PwD, policymakers, care insurers, and care professionals into the effectiveness of PiB for supporting informal caregivers of PwD.

## Trial status

In March 2022, the recruitment of participants for this research began. Currently, caregivers of PwD are being recruited (about 40% in January 2023). It is expected that the last participant will be recruited in May 2024 and that the last follow-up will be conducted in May 2026. This research protocol is based on the protocol submitted for ethical approval. Because of limited resources (time), the current research protocol paper was developed after the start of recruitment.

## Supplementary Information

**Additional file 1.** SPIRIT Checklist.pdf.

## Data Availability

The data of this study will be made available upon reasonable request.

## Abbreviations

ADL: Activities of daily living
CSES: Caregiver Self-Efficacy Scale
eCRF: Electronic case report forms
EDIZ: Perceived Stress by informal caregiving
GAIN: Gain in Alzheimer care Instrument
GLM: Generalized linear model
HADS: Hospital and Anxiety Depression Scale
ICECAP-O: Investigating Choice Experiments for the Preferences of Older People
ICER: Incremental cost-effectiveness ratio
ICMJE: International Committee of Medical Journal Editors
ICUR: Incremental cost-utility ratio
ITT: Intention-to-treat
MAR: Missing at random
NZA: Nationaal Zorg Autoriteit
PiB: Partner in Balance
PwD: Person with dementia
QALY: Quality-adjusted life-years
QDRS: Quick Dementia Rating System
QOL-AD: Quality of Life in Alzheimer’s Disease scale
RCT: Randomized controlled trial
RUD: Resource Utilization in Dementia
SPIRIT: Standard Protocol Items: Recommendations for Interventional Trials
TOPICS-MDS: The Older Persons and Informal Caregivers Survey Minimum DataSet
VAS: Visual analog scale
